# A note on dairy cow behavior when measuring enteric methane emissions with the GreenFeed emission monitoring system in tiestalls

**DOI:** 10.3168/jdsc.2023-0451

**Published:** 2023-11-17

**Authors:** Olivia A. Smith, Christina M. Rochus, Christine F. Baes, Nienke van Staaveren

**Affiliations:** 1 Centre for Genomic Improvement of Livestock, Department of Animal Biosciences, University of Guelph, Guelph, Ontario, N1G 2W1, Canada; 2 Institute of Genetics, Vetsuisse Faculty, University of Bern, Bern 3001, Switzerland; 3 Department of Population Health Sciences, Faculty of Veterinary Medicine, Utrecht University, 3584 CM Utrecht, the Netherlands

## Abstract

Changes in the environment or novel procedures can result in altered cow behavior during data collection; training is often recommended to ensure accurate data is being recorded. Currently, little is known regarding the habituation of dairy cows during methane emission testing with the GreenFeed emission monitoring system (C-Lock Inc., Rapid City, SD), or how behavior relates to enteric methane emission measurements. Methane emissions were estimated from a total of 202 Holstein dairy cows (120–150 d in milk) housed in tiestalls as part of a larger project. Cows were tested on d 0 (training day) and d 1–5 (test day) for approximately 10 min, during which behavior was recorded by a trained observer. While cows spent more time with their head outside of the machine on the training day (d 0) than during the test days (d 1–5), the opposite pattern was observed for the number of leg movements. No differences in estimated methane production were found over the different days, though it was negatively correlated with both behaviors. These results highlight the importance of habituation of dairy cows to the GreenFeed system for methane measurements to minimize changes to cow behavior under tiestall conditions, whereas the methane emissions themselves are less affected. However, further research is needed to determine the impact of cow behavior on the reliability and repeatability of methane emission measurements as it may introduce bias in genetic evaluations for methane efficiency.

## Highlights

• We investigated behavior and methane emissions in tethered cows.
• Cows show minor behavioral changes when training to measure methane emissions.
• These behaviors (head position, leg lifts) are correlated with methane production.
• Methane production was similar during training and test sessions.
• Accounting for dairy cow behavior when collecting data is important.

## Graphical Abstract

**Figure:**
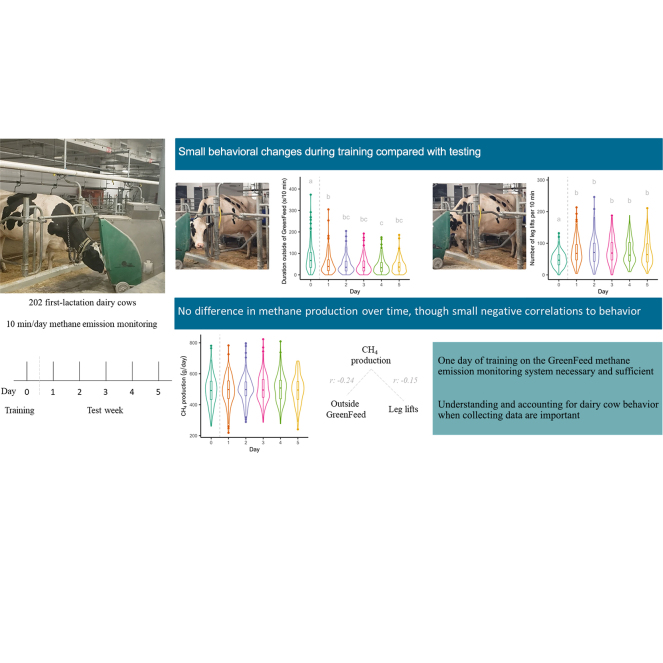
**Summary** Collection of data through technology may influence cow behavior, and behavioral changes in cows can influence data collected. For these reasons, it is often recommended that a training period for dairy cows is used before data collection starts. We have been collecting data on methane emissions in dairy cows (n = 202) using the GreenFeed methane emission monitoring system (C-Lock Inc., Rapid City, SD) and investigated whether behavior is different during the training session (day 0) compared with actual test sessions (days 1-5). We found small behavioral differences occurred, where cows spent more time with their head outside of the monitoring system and less time moving their legs during the training session. These behaviors were correlated with measured methane production, but overall methane production was the same over the training and testing sessions.

Novel procedures can lead to changes in cow behavior. Examples of this include exposure to milk training (Jago and Kerrisk, 2011), introduction to novel feed bins (Soonberg et al., 2019), and adjustment to tiestalls (Enriquez-Hidalgo et al., 2018). When novel procedures are introduced without appropriate habituation or training, an increased variability in data collected can occur and thus may lead to misleading interpretations (Rudeck et al., 2020). In dairy breeding there is a need for large amounts of accurate phenotypes to perform genetic evaluations for complex traits such as those related to methane emissions (Baes et al., 2021; Manzanilla-Pech et al., 2021). Methane emissions can be collected through different technologies (ICAR, 2020); however, to date, there has been limited research on behavior in relation to methane emissions in cattle. Earlier studies have mainly focused on feeding behavior or rumination as this plays a direct role in the fermentation process, of which methane is a byproduct (Ricci et al., 2014; López-Paredes et al., 2020). The technology used to measure methane emissions may also influence cow behavior as certain procedures may require more or less animal handling or changes in their normal routine (ICAR, 2020). This is particularly the case in respiratory chambers (Llonch et al., 2016), and less so with SF_6_ tracer techniques (Pereira et al., 2021); however, this is not often quantified. Finally, the cow's temperament itself has also been suggested to be associated with difference in methane emissions (Marçal-Pedroza et al., 2021). Understanding cow behavior and the need for habituation or behavioral components to be taken into account when modeling may lead to more accurate estimates of methane emissions (Llonch et al., 2016). To the best of our knowledge, no published studies have examined behavior of dairy cows when using the GreenFeed emission monitoring system or how behavior relates to the methane measurements collected during the test.

Data were collected at the Ontario Dairy Research Centre (Ponsonby, Ontario, Canada) from October 16, 2020, to September 30, 2022, as part of the larger Resilient Dairy Genome Project (Baes et al., 2021; van Staaveren et al., 2024). The study was approved by the University of Guelph Animal Care Committee (Animal Utilization Protocol 4445). A total of 202 first lactation Holstein dairy cows (120–150 DIM) were moved in groups of one to 4 animals from the loose housing barn to a tiestall barn (Friday morning before testing week) for individual methane emission testing. In the case only one cow was being tested, a second cow was kept in the adjacent tiestall as a social companion. A detailed description of the general housing and methane testing protocol can be found in Kamalanathan et al. (2023) and van Staaveren et al. (2024). In brief, methane testing occurred on the training day (d 0) during which cows were first exposed to the process by observing cows from the previous test week undergo methane testing, and then being tested themselves once at noon (1200 h) (Friday before testing week). The cow was then retested during the test week (d 1–5, Monday through Friday) at 0800, 1200, and 1600 h. Cows were tested in the same order on each day. A GreenFeed system (GreenFeed; C-Lock Inc., Rapid City, SD) was pushed in front of the cow and she had to place her head in the semi-enclosed head hood for methane emission measurement. Infrared sensors in the GreenFeed system detected the cow's radiofrequency identification (**RFID**) tag for individual identification when her head was properly within the hood. To ensure sufficient time to measure methane emissions, the test lasted approximately 10 to 12 min whereby the head of the cow had to remain within the machine for at least 10 min. Bait feed, consisting of familiar concentrate pellets, was dropped into the machine approximately every 23 s to entice the cow to remain inside the machine. The daily methane production (g/d) was estimated through the GreenFeed system based on gas concentration and airflow measurements for each cow while her head was inside the hood. During each test, the cow's behavior (Table 1) was recorded using continuous focal sampling. For practical reasons, the 0800 and 1200 h tests were performed by the same trained technician, and the 1600 h tests were performed by a rotating team of trained volunteers. Due to the single measurement on d 0 (1200 h) and the variability in testers at 1600 h, only measurements from 1200 h from each day for both behavior and methane emissions were included in the analysis. Thus, the daily methane production (g/d) that was extracted from the GreenFeed machine was based on one test (1200 h) which reflected the same time frame as the behavior data.

**Table 1:** Ethogram for behaviors recorded during GreenFeed (C-Lock Inc.) methane testing in Holstein dairy cows^1^

Behavior	Description
Head outside of the machine	Cow pulls muzzle out of the GreenFeed machine for >2 s. Duration is recorded (s).
Leg lift	Cow lifts leg fully from the ground. Leg can return to similar position (lift) or a different position (step) in any direction.
Leg over curb	Cow lifts front leg and places it over the curb toward GreenFeed. Lifts where the leg is moved over the curb but not fully placed down over the curb are included. Count only steps or attempts over the curb, not when moving the leg back (i.e., the leg has to be on the stall floor in the starting position).
Stomp or kick	A front leg stomp was defined as a raising of either front leg (while not walking) followed by a forceful thrust back to the ground. A hind leg kick occurred if either hind leg was thrust upward toward the animal's belly.
Tail swish bout	Tail is moved from its resting position to one side of the cow's body at the minimal height of the belly or side. A new bout is recorded if >3 s has passed between successive tail movements; otherwise, it is recorded as one occurrence.
Head shake bout	Rapid movement of the head around to the side or up/down. It does not include slow head movements in response to a visual stimulus. This behavior can only be recorded when the cow's head is outside of the machine. A new bout is recorded if >3 s has passed between successive head movements; otherwise, it is recorded as one occurrence.
Machine push	Cow pushes against GreenFeed machine with her head from inside or outside the machine.
Vocalization	Vocalization of the cow. If the sound is interrupted by >3 s of silence, this is this is recorded as separate vocalizations.
Defecation	Defecation or urination.

1 The behaviors were recorded for approximately 10 min as the cow was tested for methane emissions.

Cows that did not have complete data (e.g., due to failure in reading the RFID tag, incomplete test period, or missing records for either behavior or methane) were excluded, leaving a final 150 cows (experimental unit) with behavior and methane emissions for d 0 to 5 in the analysis. To adjust for differences in testing duration, the total duration or frequency of the recorded behaviors was divided by total test duration in minutes and then expressed per 10 min to ensure equal comparison. Many behaviors were infrequently observed (Table 2), and thus only the behaviors of having the head outside of the machine and the number of leg lifts were analyzed further. All analyses were performed using R Statistical Software v.4.2.2 (R Core Team, 2022) and the *nlme* package (Pinheiro et al., 2022). For the duration the cow had her head outside of the machine and the number of leg lifts, generalized least squares models were used to examine the effect of day on the behavior accounting for repeated measures on each cow. A similar model was performed for methane production. Assumptions of normally distributed residuals and homogeneity of variance were examined graphically and data were transformed where necessary. A Bonferroni adjustment was used to account for multiple comparisons. Results are presented as least squares means ± standard error, unless stated otherwise. Finally, Pearson correlation coefficients between behavior (head outside of machine, leg lifts) and methane production were estimated using the cor.test function from the *stats* package in R. Statistical significance was considered at *P* < 0.05 and tendencies are reported at 0.05 ≤ *P* ≤ 0.1.

**Table 2:** Descriptive statistics of the behavior and methane emission data collected during GreenFeed (C-Lock Inc.) methane testing in Holstein dairy cows (n = 150)^1^

Variable	Mean	SD	Minimum	Maximum
Head outside of the machine (s/10 min)	50.65	45.28	0	373.64
Leg lift (frequency/10 min)	70.53	39.22	0	246.15
Leg over curb (frequency/10 min)	1.19	3.28	0	68.00
Stomp or kick (frequency/10 min)	0.37	1.20	0	12.50
Tail swish bout (frequency/10 min)	4.47	7.67	0	56.67
Head shake bout (frequency/10 min)	0.12	0.66	0	16.00
Machine push (frequency/10 min)	0.09	0.61	0	10.00
Vocalization (frequency/10 min)	0.00	0.03	0	0.83
Defecation (frequency/10 min)	0.04	0.48	0	8.18
CH_4_ production (g/d)	498.01	90.54	219	823

1 The behaviors were recorded for approximately 10 min as the cow was tested for methane emissions.

Both the time that cows spent with their head outside of the machine (*F*_5,906_ = 28.9, *P* < 0.001, Figure 1A) and the number of leg lifts (*F*_5,906_ = 29.8, *P* < 0.001, Figure 1B) differed over time. Cows spent nearly double the amount of time with their head outside of the machine on d 0 compared with d 1–5 (all pairwise comparisons *P* < 0.001), while time spent outside of the machine remained relatively constant throughout the test days. Only on d 4 did cows spent less time in the machine than during d 1 (*t*_758_ = 3.0, *P* = 0.0413, Figure 1A); however, this difference was no longer present on d 5. The average methane production did not differ between the days (*F*_5,906_ = 1.280, *P* = 0.2704, Figure 1C). Finally, the amount of time animals spent with their head outside of the machine was not correlated with the number of leg lifts during testing (r = 0.04, *P* = 0.23). However, the amount of methane produced was negatively correlated with the amount of time spent outside of the machine (r = −0.23, *P* < 0.001) and the number of leg lifts (r = −0.15, *P* = 0.03).

**Figure 1:**
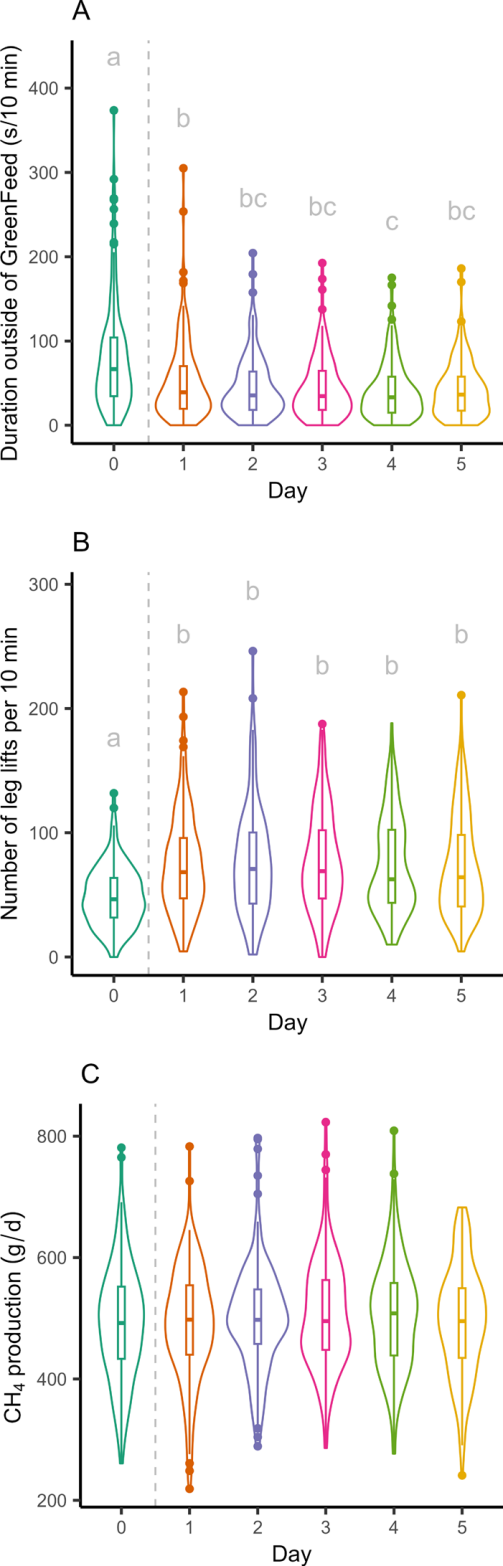
(A) Time in seconds that the cow has her head outside of the GreenFeed emission monitoring system (C-Lock Inc.) per 10 min, (B) number of leg lifts per 10 min, and (C) methane (CH_4_) production (g/d), throughout the training (d 0) and testing days (d 1–5) for enteric methane emission testing in dairy cows (n = 150). Dashed vertical line indicates the transition from training to testing days. Different letters indicate significant differences (*P* < 0.05) between days. Data shown are the observed data. Violin plots (outline) show the distribution of data per day. Boxplots indicate the median (horizontal line) and interquartile range (boxes) with whiskers extending 1.5 times the interquartile range. Dots indicate data points beyond the end of the whiskers.

Our results showed small behavioral alterations during the training session (d 0) compared with each of the testing sessions (d 1–5), whereas little to no differences were observed between the testing sessions (d 1–5) themselves. This suggests that one training session may be sufficient in terms of behavioral adaptation; however, it should be noted that cows underwent additional tests during d 1–5 (tests at 0800 and 1600 h). As such, it is likely more correct to state that a minimum of 2 training sessions are needed. The finding that cows spent more time with their head outside of the machine on d 0 is not unexpected. Cows show a greater hesitation to enter novel situations (Jago and Kerrisk, 2011) and likely the cows in the current study still needed to learn that feed was available in the machine. Contrary to our expectation, the number of leg lifts on d 0 was significantly lower than during the test sessions (d 1–5). Stepping during milking is considered to be more frequent in nervous or anxious cows (Metz-Stefanowska et al., 1992), or a sign of agitation or discomfort (Grandin, 1993; Rousing et al., 2004). However, cows have also been found to show oppositive reactions (e.g., increase or decrease in stepping behavior) in response to aversive handlers (Rushen et al., 1999; Munksgaard et al., 2001) or stress depending on their temperament (Marçal-Pedroza et al., 2021). As such, it has been suggested that the amount of movement may be situation specific rather than a general sign of fear (Munksgaard et al., 2001), and interpretation depends on the context. The cows included in our study may have been predisposed to respond to novel situations with decreased leg movements (as seen on d 0), or perhaps the increase in leg movements (as seen in d 1–5) is associated with increased anticipatory behavior for the bait feed (Neave et al., 2021). It should be acknowledged that there were some limitations in the current study where cows were followed only for a short duration within tiestall housing and acted as their own control. It would have been interesting to measure the cows' behavior before entering the tiestall area (i.e., a baseline), to allow a longer time to adjust to the tiestall itself, or to include a proper control group of cows not tested with the GreenFeed system; however, this was not feasible due to practical constraints. As such, it should be kept in mind that the comparison of cow behavior during the training session versus the testing sessions is relative and not necessarily reflecting their “normal” behavior, merely their response during the 2 phases which includes the move to the tiestall as well as the training on the GreenFeed system. The move to the tiestall environment relatively shortly before the training session on d 0 (approximately 3–4 h before) should also be acknowledged, as ideally one would have cows get accustomed to the new environment to eliminate any confounding between habituation to the tiestall and the methane testing. However, logistically this was the best option to move the cows to the tiestall and allow them to observe a testing session from the cows from the previous group, before being trained themselves. It should be noted that cows in this research herd were well accustomed to being tethered and had several hours to days (for the training and testing sessions, respectively) to adjust to the tiestall. We believe that the actual procedure of testing with the unfamiliar GreenFeed system overruled any potential impact of transfer to a different location in the barn.

Finally, cows were tested in tiestall housing, which is still the predominant housing system in Ontario (Canadian Dairy Information Centre, 2022), though steps are being taken to phase out continuous tethering in Canada by 2027 (National Farm Animal Care Council, 2023). The findings from this study should therefore not be generalized to loose housing systems where visits to the GreenFeed machine are voluntary. The voluntary number of visits to the GreenFeed machine could have been an additional indicator to consider in assessing the interaction between the animal and the machine (Della Rosa et al., 2021).

The small behavioral changes observed in the current study are in line with findings by Pereira et al. (2021) who found that cows fitted with SF_6_ equipment did not show major behavioral changes. However, in that study the actual methane emission measurements were not investigated. We found no differences in methane production across the training session and any of the test sessions, suggesting that the small behavioral changes observed did not impact the data collection for methane emissions. The behaviors observed may not have a strong biological influence on methane emissions as opposed to other behaviors such as rumination, however due to the test design this was not a behavior we could include. Moreover, the relationship between rumination time and methane emissions is also not straightforward (Zetouni et al., 2018; López-Paredes et al., 2020). The lack of difference in methane production on d 0 compared with d 1–5 could suggest the inclusion of this data point to enlarge the reference population for methane-related traits (Manzanilla-Pech et al., 2021). However caution is needed as small, but significant (*P* < 0.05), negative correlations were found between methane production and the time the cow spent with her head outside of the machine and the number of leg movements. Previous research also found differences in methane emissions as measured in respiratory chambers depending on the reactivity level of dairy cows (Marçal-Pedroza et al., 2021); however, this can also differ depending on which temperament test was used to assign the reactivity profile. More directly related to behavior performed by the cows, they found that cows that were more restless (e.g., took more steps) in the respiratory chamber tended to show a higher methane intensity (Marçal-Pedroza et al., 2021). Finally, it is important to note that the presented correlations were phenotypic and we do not know how behavior-related traits correlate to methane traits on a genotypic level. Further research to understand the relationship between behavior and methane traits is needed as it may introduce bias in genetic evaluations for methane efficiency.

In summary, the protocol of having 2 training sessions appears to be necessary and sufficient for habituation to the GreenFeed system in terms of cow behavior in tiestall conditions. Despite minor changes observed in cow behavior, no differences were observed in methane production, providing confidence in the data collected through the GreenFeed system. We found low correlations between behavior and methane production; however, these require further investigation. These results highlight the importance of taking behavior into consideration when testing cows for methane emissions.
